# Probiotic effect in preterm neonates with sepsis - A systematic review protocol

**DOI:** 10.12688/f1000research.122226.1

**Published:** 2022-08-08

**Authors:** Faiza Iqbal, N Siva, Manasa Kolibylu Raghupathy, Leslie Edward S Lewis, Apurv Barche, Jayashree Purkayastha, Baby S Nayak

**Affiliations:** 1 Department of Paediatrics, Kasturba Medical College, Manipal, Manipal Academy of Higher Education, Manipal, Karnataka, 576104, India; 2 Department of Child Health Nursing, Manipal College of Nursing, Manipal Academy of Higher Education, Manipal, Karnataka, 576104, India; 3 Department of Physiotherapy, Manipal College of Health Professions, Manipal Academy of Higher Education, Manipal, Karnataka, 576104, India

**Keywords:** Probiotics, Preterm, Neonatal Sepsis, Gut health, Mortality, Morbidity, Growth

## Abstract

**Background:** The microbiota in the intestine is made up of trillions of living bacteria that coexist with the host. Administration of antibiotics during neonatal infection causes depletion of gut flora resulting in gut dysbiosis. Over the last few decades, probiotics have been created and promoted as microbiota management agents to enrich gut flora. Probiotics decrease the overgrowth of pathogenic bacteria in the gut of preterm neonates, reducing the frequency of nosocomial infections in the Neonatal Intensive Care Unit (NICUs).

**Methods:** The systematic review will include randomized control trials (RCTs) of premier neonates with sepsis. Studies will be retrieved from global databases like Cochrane CENTRAL, CINAHL Plus via EBSCO host, MEDLINE via PubMed, EMBASE, SCOPUS, Ovid, Web of Science, ProQuest Medical Library, Microsoft academic, and DOAJ by utilizing database-specific keywords. Screening, data extraction, and critical appraisal of included research will be carried out separately by two review writers. Findings will be reported in accordance with the PRISMS-P 2020 guidelines.

**Conclusions:** The findings of this systematic review will help to translate the evidence-based information needed to encourage the implementation of potential research output in the field of neonatal intensive care, guide best clinical practise, assist policy making and implementation to prevent gut dysbiosis in neonates with sepsis by summarising and communicating the evidence on the topic.

**PROSPERO registration number:** This systematic review protocol has been registered in PROSPERO (Prospective Register of Systematic Reviews) on 10 ^th^ March 2022. The registration number is CRD42022315980.

## Introduction

According to the Global Burden of Disease (GBD), there are 1.3 million yearly incident cases of neonates sepsis and associated infections (about 937 instances per 100,000 live births) and 203,000 sepsis-related neonatal deaths. ^1^ A preterm infants’ immature intestine predisposes it to infection and inflammation, as it has immature immunity, barrier function, and peristalsis resulting in gut dysbiosis. ^2^ Co-morbidities like c-section deliveries, antenatal and post-natal antibiotic exposure, parenteral nutrition and prolonged stays in a neonatal intensive care unit (NICU), lead to a rapid decrease in taxonomic richness and diversity of good commensal gut bacteria contributing to the loss of colonization resistance. ^3^^,^^4^

The gut is the centre of microbial activity; it is essential to address neonatal microbiota dysbiosis as many of the events like period of gestation, method of delivery, dietary patterns, weaning and antibiotic administration play an important role in framing gut flora and may have an impact on long term health outcomes. ^5^^,^^6^ The host and gut microbiota have unique and cryptic interlinkage; the formation of an individual’s gut microbiota starts right from birth and is shaped by various factors. Starting from birth, the gut microbiota plays three critical roles: protective, metabolic, and trophic. ^5^ Gut flora carries out their effects by modulating immunologic, endocrine, and neurological pathways. ^7^

The gut also serves as a significant source of noninflammatory immune stimulators in healthy people throughout their lives. However, these beneficial health-promoting factors of the gut microbiota are not infallible and can be altered due to dysbiosis or neonatal infection. Numerous studies have shown that antibiotic consumption causes dysbiosis and disturbance of the gut microbiome in infants, children and adults. ^8^^,^^9^ Probiotics have been shown to improve the gut health of preterm infants. ^9^^–^^11^ Probiotics are defined by the World Health Organization as “live microorganisms that give a health benefit to the host when supplied in sufficient concentrations”. ^12^ Probiotics can enhance your immune system while preventing the formation of pathogenic gut bacteria. ^13^

A shift in the composite microbiota system has increased disease risk. ^6^ The use of probiotics facilitates the restoration of the gut microbial profile. It may protect high-risk neonates by preventing the movement of microbes and microbial metabolites across the mucosa, elimination of pathogenic organisms, modifying host reactions to microbial supplements and enhancing nutrition by upregulation of immune response. ^14^^,^^15^ A relative reduction in the *Bifidobacteriaceae* counts and an increase in Enterobacteriaceae and Clostridiaceae may be a good criterion to define dysbiosis in the initial months of life. ^11^^,^^16^ It can also explain gut colonization between birth, which can serve as the basis for introducing nutritional strategies targeted at the microbiota.

These nutritional interventions, based mainly on probiotics, may be used for favourably altering the gut microbiome and preventing neonatal sepsis. Dysbiosis of the gut microbiome invites the expansion of the pathobiont population, leading to sepsis in neonates. ^17^ Probiotic supplementation aims to rebuild the gut bacteria, preventing necrotizing enterocolitis (NEC), infection and other problems. The initial days of life are crucial for an adequate microbiome that facilitates gut maturation and neonatal health. Therefore, strategies focused primarily on establishing microbiota and consuming probiotic supplements that can help balance the gut microbiota composition, improve well-being, and lower disease risk in neonates.

### Description of the intervention?

Probiotics supplements help revive the disrupted gut microbiota and prevent gut inflammation and other intestinal diseases. ^18^ Probiotics decrease the overgrowth of pathogenic bacteria in the gut of preterm neonates, reducing the frequency of nosocomial infections in the Neonatal Intensive Care Unit (NICU). ^19^ According to systematic evaluations of randomized controlled trials (RCT), probiotics have substantial potential to lower mortality and morbidity in premature neonates. A systematic assessment of RCT narrated the benefits of probiotics in low- and middle-income countries. ^11^ The supplements may restore the composition of the gut microbiome and introduce beneficial functions to gut microbial communities. According to recent randomized controlled research, early probiotic use may have the capacity to alter the gut flora during antibiotic treatment recovery. ^20^

Probiotics are becoming more well acknowledged as a valuable tool for promoting health and preventing adverse health outcomes in premature neonates. ^21^^,^^22^ They may protect high-risk neonates by increasing the barrier for migration of bacteria and their products across the mucosa, by elimination of potential pathogens, by modifying host response to microbial products, and enhancing nutrition by upregulation of the immune response. ^23^ This study aims to evaluate the effect of probiotics on the gut health and growth of preterm infants in neonates with sepsis. We hypothesized that the administration of specific probiotics could improve the gut microbiota of preterm neonates by lowering the risk of morbidity and mortality with sepsis.

### How might the intervention work?

Over the last few decades, probiotics have been created and promoted as microbiota management agents to enrich gut flora. Early life is a crucial time for the gut of neonates to be gradually colonised with different species of bacteria that collectively promote initial gut maturation. Bacterial diversity in preterm neonates is low and comparatively different from term neonates. ^18^ Probiotic supplements may help to restore the gut microbiome composition and introduce beneficial functions to gut microbial communities, resulting in the improvement or prevention of inflammation of the gut and other phenotypes of systemic or intestinal diseases. ^24^

Probiotic supplement in preterm has been proven to decrease pathogen overgrowth and promote the maturation of the intestinal mucosa by facilitating the growth and proliferation of probiotic bacteria in the intestinal tract. ^25^ Antibiotic exposure was observed to significantly alter gut microbiota, with a considerable reduction in *Bifidobacterium* and *Lactobacillus*, according to Zhong *et al*., 2021. ^20^ The administration of probiotics concurrently with antibiotics was more advantageous to the gut microbiota than waiting until after antibiotic therapy to use probiotics, especially in terms of increasing *Bifidobacterium* abundance. ^20^ Pneumonia and sepsis accounted for 46% of all admission diagnoses and were attributed to 29% of the overall death rate, compared to 26% globally. ^26^

The mode of delivery influences the gut microbial pattern. Several high-quality studies suggest that bacterial diversity differs among babies based on the mode of delivery. Neonates born by vaginal delivery swallow the vaginal bacteria on their way down the birth canal, which results in the primary source for initial seeding of microbiota to the neonatal gut. Over the initial year of life, there is an abundance of *Bifidobacterium* spp. and a decrease of *Enterococcus* spp. and *Klebsiella* spp. ^27^ Microbiota of caesarean section neonates show distinctly different gut flora than vaginally delivered infants and are more likely to have skin, breastmilk, oral and environmental bacteria. ^28^

### Why it is important to do this review

Infections spread rapidly in preterm infants, resulting in severe disease and death; therefore, infection prevention directly reduces neonatal morbidity and mortality. In a systematic review conducted by Balasubramanian *et al*. among preterm infants in India, metanalysis showed a significantly lower risk in blood culture positive Late-Onset Sepsis (LOS) after 48 hours of birth in the probiotic group (p < 0.001). ^22^ A double-blinded, placebo-randomized controlled trial was conducted using *Bacillus clausii* to prevent LOS in 244 preterm infants. Of that, 120 were extremely preterm neonates, of which 59 received a placebo and 61 received probiotics. On other hand, 124 babies were stratified as preterm, 61 neonates received a placebo and 61 received probiotics. The study concluded that prophylactic administration of *B. clausii* to preterm neonates did not result in a significant difference in LOS incidence compared with placebo. ^29^

The gut microbiome is a complex and dynamic population of hundreds of bacteria responsible for transporting nutrition, controlling intestinal epithelial maturation, and building an innate immune defence in neonates. An RCT was conducted by Panigrahi *et al.* to prevent sepsis among rural Indian neonates. Researchers used *Lactobacillus plantarum* plus fructooligosaccharide as a probiotic on 4,556 neonates of birthweight < 2000 grams, gestational age ≥ 35 weeks with no sign of sepsis and morbidity and were recruited and monitored for 60 days; a significant decrease in neonatal sepsis (culture-positive sepsis, culture-negative sepsis) and lower respiratory tract infections were observed. The study’s findings suggested that neonatal sepsis in developing countries could be effectively prevented using a symbiotic containing *L. plantarum* ATCC-202195. ^30^ The findings of this systematic review would definitively answer whether early administration of specific probiotics could improve the gut microbiota of preterm neonates by lowering the risk of infant morbidity and mortality in neonates with sepsis, and assist healthcare providers and policymakers in developing a probiotic supplement guideline for sepsis neonates.

## Methods

### Ethical considerations

As this is a systematic review protocol, ethical approval is not required as we will not be directly involving human participants.

### Review questions

How effective is probiotics on gut health, lowering the risk of morbidity and mortality among preterm neonates with sepsis?

### Specific objectives

To identify the effect of probiotics on improving gut health of preterm neonates with sepsis.

To determine the impact of probiotics on lowering the risk of morbidity and mortality among preterm neonates with sepsis.

### Design

We will systematically review existing randomized and non-randomized control trial studies and execute a meta-analysis when acceptable data is available. This protocol follows the recommendations of the Preferred Reporting Items for Systematic Review and Meta-Analyses of Protocols (PRISMA-P) 2015. ^40^ The Participants, Interventions, Comparators and Outcomes elements used for the systematic review are listed in Table 1.

**Table 1.** Eligibility criteria. NICU: Neonatal Intensive Care Unit; NRCT’s: Non- randomized controlled trials.

Inclusion and exclusion criteria		
PICO component	Inclusion criteria	Exclusion criteria
**Population (P)**	Preterm neonates (Gestational age <36 weeks) with sepsis.	Term neonates and preterm neonates without sepsis.
**Intervention (I)**	All types of probiotic supplements administered to preterm with sepsis.	Administered prebiotics and synbiotic supplements to preterm with sepsis.
**Comparison (C)**	Placebo or standardized NICU treatment.	Prebiotics and synbiotic supplements.
**Outcome (O)**	**Primary outcome:** Preterm neonatal gut health, morbidity, and mortality **Secondary outcome:** Weight of the baby.	
**Time (T)**	Articles published from January 2010 to April 2022 with full text in English language.	
**Study design (S)**	RCTs, NRCT’s.	Observational studies, Qualitative studies, reviews, editorials, conference abstracts, study protocols, reports, and letters.

### Search strategy

A comprehensive search would be conducted in global databases like Cochrane CENTRAL, CINAHL Plus via EBSCO host, MEDLINE via PubMed, EMBASE, SCOPUS, Ovid, Web of Science, ProQuest Medical Library, Microsoft academic, and DOAJ. by utilizing the stated search keywords: “Premature neonates”, “Preterm birth”, “Very low birth weight”, “Low birth weight”, Preterm, “Preterm infants”, “extremely low birth weight”, “Neonates”, “Neonatal infection”, Infection*, “Late-onset sepsis”, “Early-onset sepsis”, Sepsis, “Neonatal period”, “intestinal infection”, “Nosocomial infection”, septicaemia, Bacteraemia, “septic shock”, probiotic*, “Probiotic supplement”, Lactobacilli, Lactobacillus, Bifidobacterium, “Lactic acid bacteria”, Bacillus OR “Probiotic therapy”, “Randomized Controlled Trial”, and “Non- randomized controlled trial” ( *Extended data* ^38^).

### Search for other resources

Additional references will be found in the reference lists of all primary research and review papers. To get necessary supplementary information, we will reach out to relevant field experts and researchers from the included papers. For data management, records will be exported to EndNote X7.

### Selection of studies

Two review authors (FI, SN) shall execute independent searches of the study titles and abstracts of the indicated study sources. We shall exclude the articles which do not meet the inclusion criteria. If there is a disagreement among those reviewers, it will be resolved through conversation, and the complete text will be examined. Two reviewers will independently assess the included abstracts as “include” or “exclude” after obtaining the whole manuscript. If required, we will work with a supervisory review author (LEL) to address any disagreements. We will keep track of why the articles were refused. We will look for and eliminate duplicates and compile numerous reports from the same research.

### Data extraction and management

Data will be extracted by examining general features such as gestational age, research participants, setting, identifiers, type of pathogen isolated, LOS, early-onset sepsis (EOS), type of probiotic, study selection criteria, and results. To organize the list of intervention parameters to assist intervention replication and research comparability, we will use the TIDier checklist (Template for Intervention Description and Replication). ^31^ Additionally, we will extract data of specific characteristics such as type of probiotic used, dosage, antibiotic exposure, gestational age, type of delivery and trend of weight gain. Two review authors will independently use the data extraction templates to abstract the data from included articles in the review. The included studies will have the following data collected: research title and authors, sample size, study setting, intervention features, assessment procedures, outcomes, findings, conclusions, and year of publication. If there is a disagreement, we will discuss it until we reach an agreement, or we will handle it with the assistance of a third reviewer (MKR).

### Assessment of quality of the studies

We will assess the quality of included randomized controlled trials using the Revised Cochrane Risk of Bias Tool (RoB 2.0). The RoB 2.0 tool considers the following factors: a) The randomization method, b) deviation from the planned intervention, c) outcome measurement, d) missing outcomes, and e) selective reporting. Two authors will independently evaluate each paper. RCT risk will be assessed and classified into three categories: low risk, high risk, and some concerns. ^32^ To assess the quality of the included non-randomized controlled trials, we will use the Joanna Briggs Institute (JBI) Critical Appraisal Checklist. If there is any disagreement, we will solve it by contacting a methodology expert (BSN).

### Dealing with missing data

We will contact the primary authors whenever possible to obtain the missing data. The most important empirical data, including screening, randomization, intention-to-treat, as-treated, and per-protocol groups, will be investigated thoroughly. The article will be excluded from the review if the authors do not respond within two weeks after communicating through email. If this is not possible, we will consider the missing data a major bias, and the article will be removed from consideration.

### Subgroup analysis

If possible, sub-group analysis will be carried out based on the neonatal gestational age (extremely preterm, very preterm, moderate preterm and late preterm) and study period.

### Data synthesis

Two independent reviewers (FI and SN) will extract the data from each study and enter it into a Microsoft Excel file Version 16.61. Preferred Reporting Items for Systematic Review and Meta-analysis (PRISMA) standards will be used to provide evidence regarding the effect of probiotics on the gut health of preterm neonates with sepsis ( *Extended data* ^39^). ^33^ The GRADE (Grading of Recommendations Assessment, Development, and Evaluation) approach will be employed to assess the quality of evidence in the included articles. The quality of the information is rated as a four-point scale, high, moderate, low, and very low. If there is sufficient homogeneity in study design and study subjects among the selected studies, meta-analyses will be conducted. ^34^ As a result, continuous and dichotomous outcomes will be integrated for meta-analysis purposes. Statistical analyses will be conducted with the help of the Cochrane Review Manager (RevMan 5).

### Sensitivity analysis

Sensitivity analysis will be performed to investigate the robustness of the results by examining the effects of including and excluding studies with a high risk of bias and studies with missing data. The results’ robustness will be evaluated using a variety of impact size measures, such as risk ratio and odds ratio, and various statistical models, such as fixed effects and random-effects models.

## Discussion

The microbiota in the intestine is made up of trillions of living bacteria that coexist with the host. Depleting gut microflora in neonates, especially in vulnerable preterm neonates, may elevate the risk of neonatal infections resulting in administration of antibiotics. ^35^ Although antibiotics are targeted to kill only pathogens, they may adversely affect commensals by destroying them or inhibiting their activity, resulting in gut dysbiosis. Probiotic supplementation has become increasingly popular in the fight against gut microbiota depletion. ^16^^,^^20^^,^^21^

A randomized control trial was conducted by Zhong *et al.* to assess the impact of probiotic supplements on 90 neonates with gestational age ≥ 37 weeks. The study concluded that antibiotics cause a decrease in the microbial richness and variety of the gut microbiota in neonates and the attenuation of several bacteria, especially *Bifidobacterium* and *Lactobacillus.* ^20^ A significant reduction in newborn sepsis has been reported in randomized controlled trials (RCTs) of various probiotic strains and combinations given to preterm infants of different gestational ages and birth weights. Panigrahi *et al*., in their randomized control trial, examined the effect of probiotics in neonatal sepsis and showed a significant reduction in neonatal sepsis cases when supplemented with *Lactobacillus plantarum.* ^36^ Costeloe *et al.* evaluated the effect of *Bifidobacterium breve* strain BBG-001 on the development of sepsis or NEC in very low birth weight neonates. At two weeks postnatal age, he observed *B. breve* colonisation in 1186 (94%) survivors. There were 85% in the probiotic group and 37% in the placebo group. ^37^

### Strengths and limitations of the study

This systematic review will include randomized controlled trials and all types of non-randomized control trials. Meta-analysis will be carried out with the results of RCTs, and narrative description will be used to analyse non-randomized control trials regarding the effect of probiotics among preterm neonates with sepsis. This review includes only the use of probiotics in preterm infants, excluding the studies that assessed the effect of prebiotics and synbiotics among preterm neonates. Only studies published in English will be included; thus, eligible studies published in other languages will be excluded.

## Data availability

### Underlying data

No underlying data are associated with this article

### Extended data

Figshare: supplementary_file1.docx. https://doi.org/10.6084/m9.figshare.19839604. ^38^

This project contains the following extended data:

• Supplementary_File1.docx (Appendix 1- Search Strategy; Appendix 2 – Search Terms)

Figshare: Supplementary file 2. https://doi.org/10.6084/m9.figshare.19839964. ^39^

This project contains the following extended data:

• Supplementary_file2.pdf (PRISMA flow chart of Systematic review)

Data are available under the terms of the Creative Commons Attribution 4.0 International license (CC-BY 4.0).

### Reporting guidelines

Figshare: PRISMA-P checklist for ‘Probiotic effect in preterm neonates with sepsis - A systematic review protocol’, https://doi.org/10.6084/m9.figshare.19839241. ^40^

Data are available under the terms of the Creative Commons Attribution 4.0 International license (CC-BY 4.0).
