# Supplementary material for: Probiotic effect in preterm neonates with sepsis - A systematic review protocol
Source: F1000Res. 2024 Jul 5;11:913. Originally published 2022 Aug 8. [Version 3] doi: 10.12688/f1000research.122226.3 (PMC11303946; doi:10.12688/f1000research.122226.3)
Supplement: Peer review history

# Reviewer report: Reviewer response for version 3

**DOI:** 10.5256/f1000research.168360.r306773

**Published:** 2024-08-06

**Authors:** Yao Wang

**Affiliations:** 1 The Chinese University of Hong Kong, Hong Kong, Hong Kong

Sepsis is a severe condition that threatens fetal health. Applying probiotics is a feasible and potential approach to prevent this disease. Therefore, this study is meaningful with significant clinical implications. The methods and study design are clear and reasonable. Importantly, the authors will extract specific data such as the type of probiotic used, dosage, antibiotic exposure, gestational age, and type of delivery.

 One suggestion is that the details of probiotics should be clearly documented at the species or strain level if the data allows. Moreover, the prescribed model, including the starting point of administration during pregnancy and the duration of probiotic use, should also be included. All these contents are crucial to help clinicians to understand and make decisions. An example study is Bekalu et al., AJOG FMF 2023. 10.1016/j.ajogmf.2023.101148.

 I am overall content if the authors could further improve above comments.

Is the study design appropriate for the research question?

Yes

Is the rationale for, and objectives of, the study clearly described?

Yes

Are sufficient details of the methods provided to allow replication by others?

Yes

Are the datasets clearly presented in a useable and accessible format?

Yes

Reviewer Expertise:

Microbiome, Maternal-fetal health, Exercise Intervention, RCT,

I confirm that I have read this submission and believe that I have an appropriate level of expertise to confirm that it is of an acceptable scientific standard.

## References

[1] : Effects of maternal probiotic supplementation on breast milk microbiome and infant gut microbiome and health: a systematic review and meta-analysis of randomized controlled trials. *Am J Obstet Gynecol MFM* .2023;5(11) : 10.1016/j.ajogmf.2023.101148 101148 10.1016/j.ajogmf.2023.101148 37660760

# Reviewer report: Reviewer response for version 2

**DOI:** 10.5256/f1000research.157165.r278782

**Published:** 2024-06-13

**Authors:** Sabina Fijan

**Affiliations:** 1 Faculty of Health Sciences, University of Maribor, Maribor, Slovenia

**Probiotic effect in preterm neonates with sepsis - A systematic review protocol: Review report**

 This manuscript, submitted to the journal f1000Research, is a crucial topic and describes the protocol to conduct a systematic review of the probiotic effect in preterm neonates with sepsis.

**General comments**

 Recent systematic reviews on this subject have been published and should be referenced with the rationale for conducting similar research (Kulkarni et al., 2022; Thomas et al., 2023).

 Probiotics are by definition: **“live microorganisms that, when administered in adequate amounts, confer a health benefit on the host”** (Hill et al., 2014). This definition is published as a consensus statement by the International Scientific Association for Probiotics and Prebiotics and should be referenced when citing the correct definition. This definition is in line with the definition accepted by the Food and Agriculture Organization of the United Nations and the World Health Organisation (FAO/WHO) published in 2001 (FAO/WHO, 2001) and the guidelines to assist with this definition published in 2002 (FAO/WHO, 2002).

 The human microbiota comprises of bacteria, archaea, viruses, fungi and other eukaryotes that coexist in and on various sites of the human body (Hou et al., 2022), while the microbiome is the collection of all genetic information of this microbiota (Grice & Segre, 2012; Ogunrinola et al., 2020). The term ‘flora’ is outdated and should be replaced by the taxonomically correct term ‘microbiota.

 When comparing the modulation of gut microbiota, the abundance or ratio of certain phyla is usually compared (Walsh et al., 2014). However, it is necessary to use up-to-date nomenclature (Oren & Garrity, 2021). Firmicutes are now Bacillota, Proteobacteria are now Pseudomonadota, Actinobacteria are now Actinomycetota, Bacteroidetes are now Bacteroidota etc.

 Up-to-date terminology should be used for lactobacilli (Zheng et al., 2020). This genus *Lactobacillus* has been divided and now consists of 25 genera. It is necessary to include the new nomenclature of many species that contain probiotic strains, for example: *Limosilactobacillus reuteri *(previously *Lactobacillus reuteri*), *Limosilactobacillus reuteri *(previously *Lactobacillus reuteri*), *Lactiplantibacillus plantarum *(previously *Lactobacillus plantarum*), *Levilactobacillus brevis *(previously *Lactobacillus brevis*), *Lacticaseibacillus rhamnosus *(previously *Lactobacillus rhamnosus*), *Ligilactobacillus salivarius *(previously *Lactobacillus salivarius*). The new nomenclature can be found at the website: http://lactotax.embl.de/wuyts/lactotax/ . Other genera that also include probiotic strains such as *Bacillus*, *Clostridium, Propionibacterium* have also undergone taxonomic reclassification (Gupta et al., 2020; Lawson et al., 2016; Scholz & Kilian, 2016).

 Another important factor is the meaning of probiotics. Only certain strains are probiotic microbes. The strains that are labeled as a probiotic must have at least on quality double blind randomised controlled clinical study that exhibits a statistically significant difference in the group that was supplemented with probiotics compared to the placebo group (Binda et al., 2020). And in many instances the health benefits are strain-specific, therefore strain information must be included. For example, *Lacticaseibacillus rhamnosus* GG (previously known as *Lactobacillus rhamnosus* GG) is a well-known probiotic with several proven health benefits (McFarland et al., 2021; Tan et al., 2021). However, this does not mean that all strains of the species *Lacticaseibacillus rhamnosus* are probiotics, let alone all lactobacilli. The same applies for *Limosilactobacillus reuteri* strains, *Lactiplantibacillus strains*, and many others.

**Minor corrections**

- Introduction, paragraph 3: please correct the sentence to ‘probiotics can enhance your immune system while preventing the growth pathogenic gut bacteria'.
- Assessment of quality of the studies: please add the foreseen Joanna Briggs Institute Critical appraisal tool checklist for randomised controlled trials.
- Discussion: paragraph 1: please use accurate phrases. antibiotics are pharmacological agents that destroy the bacteria cells (Patel P et al., 2023), but not viruses that are also possible gut pathogens.
- Discussion: paragraph 1: some connective sentences should be included, not just the presentation of results of randomised controlled trials. When referring to the conductors of clinical trials please use the term ‘they’.

Is the study design appropriate for the research question?

Yes

Is the rationale for, and objectives of, the study clearly described?

Yes

Are sufficient details of the methods provided to allow replication by others?

Yes

Are the datasets clearly presented in a useable and accessible format?

Not applicable

Reviewer Expertise:

probiotics, beneficial microbes, fermented foods, health benefits

I confirm that I have read this submission and believe that I have an appropriate level of expertise to confirm that it is of an acceptable scientific standard, however I have significant reservations, as outlined above.

## References

[1] : Criteria to Qualify Microorganisms as. *Front Microbiol* .2020;11: 10.3389/fmicb.2020.01662 1662 10.3389/fmicb.2020.01662 32793153 PMC7394020

[2] Health and Nutritional Properties of Probiotics in Food including Powder Milk with Live Lactic Acid Bacteria. Food and Agriculture Organization of the United Nations, World Health Organization. *FAO/WHO* .2001;

[3] Guidelines for the Evaluation of Probiotics in Food. *FAO/WHO* .2002;

[4] : The human microbiome: our second genome. *Annu Rev Genomics Hum Genet* .2012;13: 10.1146/annurev-genom-090711-163814 151-70 10.1146/annurev-genom-090711-163814 22703178 PMC3518434

[5] : Robust demarcation of 17 distinct Bacillus species clades, proposed as novel Bacillaceae genera, by phylogenomics and comparative genomic analyses: description of Robertmurraya kyonggiensis sp. nov. and proposal for an emended genus Bacillus limiting it only to the members of the Subtilis and Cereus clades of species. *Int J Syst Evol Microbiol* .2020;70(11) : 10.1099/ijsem.0.004475 5753-5798 10.1099/ijsem.0.004475 33112222

[6] : Expert consensus document. The International Scientific Association for Probiotics and Prebiotics consensus statement on the scope and appropriate use of the term probiotic. *Nat Rev Gastroenterol Hepatol* .2014;11(8) : 10.1038/nrgastro.2014.66 506-14 10.1038/nrgastro.2014.66 24912386

[7] : Microbiota in health and diseases. *Signal Transduct Target Ther* .2022;7(1) : 10.1038/s41392-022-00974-4 135 10.1038/s41392-022-00974-4 35461318 PMC9034083

[8] : Probiotic sepsis in preterm neonates-a systematic review. *Eur J Pediatr* .2022;181(6) : 10.1007/s00431-022-04452-5 2249-2262 10.1007/s00431-022-04452-5 35348825

[9] : Reclassification of Clostridium difficile as Clostridioides difficile (Hall and O’Toole 1935) Prévot 1938. *Anaerobe* .2016;40: 10.1016/j.anaerobe.2016.06.008 95-99 10.1016/j.anaerobe.2016.06.008 27370902

[10] : Specific Probiotics for the Treatment of Pediatric Acute Gastroenteritis in India: A Systematic Review and Meta-Analysis. *JPGN Rep* .2021;2(3) : 10.1097/PG9.0000000000000079 e079 10.1097/PG9.0000000000000079 37205949 PMC10191489

[11] : The Human Microbiome and Its Impacts on Health. *Int J Microbiol* .2020;2020: 10.1155/2020/8045646 8045646 10.1155/2020/8045646 32612660 PMC7306068

[12] : Valid publication of the names of forty-two phyla of prokaryotes. *Int J Syst Evol Microbiol* .2021;71(10) : 10.1099/ijsem.0.005056 10.1099/ijsem.0.005056 34694987

[13] : The natural history of cutaneous propionibacteria, and reclassification of selected species within the genus Propionibacterium to the proposed novel genera Acidipropionibacterium gen. nov., Cutibacterium gen. nov. and Pseudopropionibacterium gen. nov. *Int J Syst Evol Microbiol* .2016;66(11) : 10.1099/ijsem.0.001367 4422-4432 10.1099/ijsem.0.001367 27488827

[14] : Lactobacillus rhamnosus GG for Cow's Milk Allergy in Children: A Systematic Review and Meta-Analysis. *Frontiers in Pediatrics* .2021;9: 10.3389/fped.2021.727127 10.3389/fped.2021.727127 PMC856990334746056

[15] : Probiotics for the prevention of mortality and sepsis in preterm very low birth weight neonates from low- and middle-income countries: a Bayesian network meta-analysis. *Frontiers in Nutrition* .2023;10: 10.3389/fnut.2023.1133293 10.3389/fnut.2023.1133293 PMC1030041937388635

[16] : Beneficial modulation of the gut microbiota. *FEBS Lett* .2014;588(22) : 10.1016/j.febslet.2014.03.035 4120-30 10.1016/j.febslet.2014.03.035 24681100

[17] : A taxonomic note on the genus Lactobacillus: Description of 23 novel genera, emended description of the genus Lactobacillus Beijerinck 1901, and union of Lactobacillaceae and Leuconostocaceae. *Int J Syst Evol Microbiol* .2020;70(4) : 10.1099/ijsem.0.004107 2782-2858 10.1099/ijsem.0.004107 32293557

[18] : StatPearls [Internet]. *StatPearls Publishing* .2023;

# Response

**Published:** 2024-06-24

**Authors:** Faiza Iqbal

**Affiliations:** Kasturba Medical College Hospital, India

Thank you for your detailed review and constructive feedback. We have addressed each of your comments as follows:

**Comment #1**: Recent systematic reviews on this subject have been published and should be referenced with the rationale for conducting similar research (Kulkarni et al., 2022; Thomas et al., 2023).

**Authors Reply:**

 Recent systematic reviews by Kulkarni et al. (2022) and Thomas et al. (2023) has referenced in the introduction to provide context and rationale for conducting similar research.

**Comment #2:**Probiotics are by definition: “live microorganisms that, when administered in adequate amounts, confer a health benefit on the host” (Hill et al., 2014). This definition is published as a consensus statement by the International Scientific Association for Probiotics and Prebiotics and should be referenced when citing the correct definition. This definition is in line with the definition accepted by the Food and Agriculture Organization of the United Nations and the World Health Organisation (FAO/WHO) published in 2001 (FAO/WHO, 2001) and the guidelines to assist with this definition published in 2002 (FAO/WHO, 2002).

**Authors Reply:**

 The definition of probiotics has been updated to include the consensus statement by Hill et al. (2014) and references to the FAO/WHO definitions from 2001 and 2002.

**Comment #3:**The human microbiota comprises of bacteria, archaea, viruses, fungi and other eukaryotes that coexist in and on various sites of the human body (Hou et al., 2022), while the microbiome is the collection of all genetic information of this microbiota (Grice & Segre, 2012; Ogunrinola et al., 2020). The term ‘flora’ is outdated and should be replaced by the taxonomically correct term ‘microbiota.

**Authors Reply:**

 The term 'flora' has been replaced with 'microbiota' throughout the manuscript.

**Comment #4:**When comparing the modulation of gut microbiota, the abundance or ratio of certain phyla is usually compared (Walsh et al., 2014). However, it is necessary to use up-to-date nomenclature (Oren & Garrity, 2021). Firmicutes are now Bacillota, Proteobacteria are now Pseudomonadota, Actinobacteria are now Actinomycetota, Bacteroidetes are now Bacteroidota etc.

**Authors Reply:**

 Up-to-date nomenclature for bacterial phyla and genera has been adopted, following the guidelines by Oren & Garrity (2021) and Zheng et al. (2020).

**Comment #5:**Up-to-date terminology should be used for lactobacilli (Zheng et al., 2020). This genus Lactobacillus has been divided and now consists of 25 genera. It is necessary to include the new nomenclature of many species that contain probiotic strains, for example: Limosilactobacillus reuteri (previously Lactobacillus reuteri), Limosilactobacillus reuteri (previously Lactobacillus reuteri), Lactiplantibacillus plantarum (previously Lactobacillus plantarum), Levilactobacillus brevis (previously Lactobacillus brevis), Lacticaseibacillus rhamnosus (previously Lactobacillus rhamnosus), Ligilactobacillus salivarius (previously Lactobacillus salivarius). The new nomenclature can be found at the website: http://lactotax.embl.de/wuyts/lactotax/. Other genera that also include probiotic strains such as Bacillus, Clostridium, Propionibacterium have also undergone taxonomic reclassification (Gupta et al., 2020; Lawson et al., 2016; Scholz & Kilian, 2016).

**Authors Reply:**

 Modified accordingly

**Comment #6:**Another important factor is the meaning of probiotics. Only certain strains are probiotic microbes. The strains that are labeled as a probiotic must have at least on quality double blind randomised controlled clinical study that exhibits a statistically significant difference in the group that was supplemented with probiotics compared to the placebo group (Binda et al., 2020). And in many instances the health benefits are strain-specific, therefore strain information must be included. For example, Lacticaseibacillus rhamnosus GG (previously known as Lactobacillus rhamnosus GG) is a well-known probiotic with several proven health benefits (McFarland et al., 2021; Tan et al., 2021). However, this does not mean that all strains of the species Lacticaseibacillus rhamnosus are probiotics, let alone all lactobacilli. The same applies for Limosilactobacillus reuteri strains, Lactiplantibacillus strains, and many others.

**Authors Reply:**

 Modified accordingly in manuscript

 Minor corrections

**Comment #7:**Introduction, paragraph 3: please correct the sentence to ‘probiotics can enhance your immune system while preventing the growth pathogenic gut bacteria'.

**Authors Reply:**

 The sentence has been corrected to "Probiotics can enhance your immune system while preventing the growth of pathogenic gut bacteria."

**Comment #8: **Assessment of quality of the studies: please add the foreseen Joanna Briggs Institute Critical appraisal tool checklist for randomised controlled trials.

**Authors Reply:**

 For the assessment of the quality of randomized controlled trials (RCTs) used in our study, we have utilized the Revised Cochrane Risk of Bias Tool (RoB 2.0), not the Joanna Briggs Institute Critical Appraisal Tool. The methodology section has been updated to reflect this, ensuring clarity on the quality assessment process used in our research.

**Comment #9:**Discussion: paragraph 1: please use accurate phrases. antibiotics are pharmacological agents that destroy the bacteria cells (Patel P et al., 2023), but not viruses that are also possible gut pathogens.

**Authors Reply:**

 The sentence has been corrected.

**Comment #10:**Discussion: paragraph 1: some connective sentences should be included, not just the presentation of results of randomised controlled trials. When referring to the conductors of clinical trials please use the term ‘they’.

**Authors Reply:**

 Connective sentences has been added for clarity, and when referring to the conductors of clinical trials, the term 'they' has been used..

 We hope these revisions will adequately address your concerns and improve the clarity and accuracy of our manuscript. Please let us know if any further modifications are required. Thank you for your valuable feedback and guidance.

# Reviewer report: Reviewer response for version 2

**DOI:** 10.5256/f1000research.157165.r217414

**Published:** 2023-10-27

**Authors:** Cecile Leah T Bayaga

**Affiliations:** 1 Department of Food Science and Nutrition, College of Home Economics, University of the Philippines, Quezon City, Philippines

The article has improved comparing it from version 1.  However, I could not follow through the general purpose of the article, matching it with my idea of what a systemic review article should be.  As I have mentioned in my earlier review, the paper would benefit by comparing the current status of the paper with a checklist on STROBE.

Reviewer Expertise:

My area of expertise is on clinical nutrition specifically chemical and microbiological composition of human milk.

I confirm that I have read this submission and believe that I have an appropriate level of expertise to confirm that it is of an acceptable scientific standard, however I have significant reservations, as outlined above.

# Response

**Published:** 2023-10-30

**Authors:** Faiza Iqbal

**Affiliations:** Kasturba Medical College Hospital, India

Dear Dr.Cecile Leah T Bayaga,

 Thank you for your feedback and for taking the time to review our systematic review protocol. We appreciate your comments and suggestions.

 We have carefully considered your previous feedback and have made efforts to improve the clarity and focus of the protocol.

 Regarding your suggestion to compare the protocol with a checklist on STROBE, we appreciate the recommendation for a structured evaluation. However, it's important to note that STROBE is primarily designed for reporting observational studies, and our protocol is focused on the systematic review protocol. Hence, we have utilized the PRISMA-P (Preferred Reporting Items for Systematic Review and Meta-Analysis Protocols) checklist, which is tailored to assess the quality and completeness of systematic review protocols.

 We have written the protocol based on the PRISMA-P checklist to ensure that it adheres to recognized standards for systematic review protocol, which is provided in Extended data under reporting guidelines.You can find the checklist at this link: 

 https://doi.org/10.6084/m9.figshare.19839241.

 Once again, we appreciate your valuable feedback, and if you have any further comments or suggestions, please feel free to share them.

 Sincerely,

 Faiza Iqbal

# Reviewer report: Reviewer response for version 1

**DOI:** 10.5256/f1000research.134189.r198892

**Published:** 2023-09-14

**Authors:** Cecile Leah T Bayaga

**Affiliations:** 1 Department of Food Science and Nutrition, College of Home Economics, University of the Philippines, Quezon City, Philippines

**General Observation**

 From my perspective, the paper is unfished. There are no results of the systematic review. The tense used is in the future; hence it will still be implemented.

**Specific Observation**

**Abstract**

 Following the PRISMA guidelines, the abstract lacks the following details:

1. Explicitly stated objective
2. Inclusion/Exclusion criteria
3. Methods on how bias was lessened
4. Methods on how data were synthesized
5. No major results and discussion
6. No mention of funding agency

**Introduction**

 First, majority of the systematic reviews published do not have subsections under this portion. Having sub sections probably is beneficial when the author writes the Introduction. However, reading is made smooth if there were no sub sections. Second, the specific objective which was supposed to be in this section was found in the Methods section. Last, the stated objective of the study does not jive with the stated hypothesis. The premise “growth of preterm infants” was included in the objective but absent in the hypothesis.

**Methods**

 There is a need to refine the methods to indicate it has been implemented, make the connection

 seamless, and revise the statements to make them clearer.

**Discussion**

 There was no discussion as there were no results presented.

Are the rationale for, and objectives of, the Systematic Review clearly stated?

Partly

Is the statistical analysis and its interpretation appropriate?

No

Are sufficient details of the methods and analysis provided to allow replication by others?

No

Are the conclusions drawn adequately supported by the results presented in the review?

No

Reviewer Expertise:

My area of expertise is on clinical nutrition specifically chemical and microbiological composition of human milk.

I confirm that I have read this submission and believe that I have an appropriate level of expertise to state that I do not consider it to be of an acceptable scientific standard, for reasons outlined above.

# Response

**Published:** 2023-09-14

**Authors:** Faiza Iqbal

**Affiliations:** Kasturba Medical College Hospital, India

Dear Reviewer,

 Thank you for taking the time to review our systematic review protocol. We appreciate your feedback and would like to address your observation that the paper appears unfinished due to the absence of results and the use of future tense.

 It's essential to clarify that a systematic review protocol is a prelude to the actual systematic review. Protocols are written before the systematic review and data extraction phases commence, and they serve as a comprehensive plan that outlines the steps and procedures that will be followed during the review process.

 The use of future tense in the protocol is intentional and necessary because, protocols are typically written before starting the search and data extraction process. At this stage, the review team has not yet gathered and analyzed the data, so it would be premature to present any results. Instead, the protocol serves as a blueprint for the systematic review, detailing the research questions, eligibility criteria, search strategy, data extraction methods, and analysis plan.

 Rest assured that once the systematic review is completed, we will publish the results in a separate paper or report, where we will present and discuss our findings based on the data collected and analyzed during the review process. This follow-up publication will provide a comprehensive overview of the results and their implications.

 We hope this clarifies the purpose and scope of our systematic review protocol. We value your input and appreciate your understanding of the process. If you have any further questions or suggestions, please feel free to share them.

 Sincerely,

 Faiza Iqbal
